# Atypical imaging presentation of a massive intracavitary cardiac thrombus: A case report and brief review of the literature

**DOI:** 10.1016/j.radcr.2021.06.089

**Published:** 2021-08-01

**Authors:** Georgia M. Vasilakis, Dhairya A. Lakhani, Ayodele Adelanwa, Jeffery P. Hogg, Cathy Kim

**Affiliations:** a School of Medicine, West Virginia University School of Medicine, WV, 26506, USA; b Department of Radiology, West Virginia University, Morgantown, WV, 26506, USA; c Section of Cardiothoracic Imaging, Department of Radiology, West Virginia University, Morgantown, WV, 26506, USA; d Department of Pathology, West Virginia University, Morgantown, WV, 26506, USA

**Keywords:** Cardiac mass, Thrombus, ITP, immune thrombocytopenic purpura, SLE, systemic lupus erythematosus, IV, intravenous, TTE, transthoracic echocardiogram, SSFP, steady state free precession, CTA, CT angiography, TEE, transesophageal echocardiogram

## Abstract

Intracavitary cardiac thrombi, uncommonly found in the right chambers, have been shown to form secondary to endocardial and myocardial diseases. The differential diagnosis for an intracavitary cardiac mass is broad, including primary cardiac tumors, cardiac metastases, anatomic variants, vegetations, and thrombi. Here we present a unique case with a large calcified intracavitary cardiac thrombus in a 26-year-old woman with obesity, immune thrombocytopenic purpura, and a new diagnosis of systemic lupus erythematosus. Initial imaging presentation in this case masqueraded as a tumor, delaying the true diagnosis. A combination of cardiac imaging techniques, including transthoracic and transesophageal echocardiograms, cardiac CT, and cardiac MRI were required to correctly diagnose this calcified bland thrombus.

## Background

Intracavitary cardiac thrombi have been shown to form secondary to endocardial or myocardial diseases. More commonly, these thrombi form from acute myocardial infarction, left ventricular aneurysm, or dilated cardiomyopathy. Uncommon causes of intracardiac thrombus formation include blunt chest trauma, systemic lupus erythematosus, and amyloidosis [1]. Risk factors that increase the likelihood of thrombus formation and embolization include cardiac structural abnormalities, flow abnormalities due to compromised ventricular contraction, valvular disease, and abnormalities of hemostasis (such as hypercoagulable states). Efforts to decrease the risk of distal embolization of the thrombus includes treatment with anticoagulation [1], [2], [3].

The most common location of an intracavitary thrombus is the left atrium, often secondary to atrial fibrillation [1] or mitral stenosis [4]. Indwelling catheters, commonly used for hemodialysis, are reported to contribute to right atrial intracavitary thrombi, as an extension of line-tip thrombosis. It has also been reported that some catheters are made from materials more thrombogenic than others [5], [6], [7]. Acute myocardial infarction can result in left ventricular wall motion abnormalities. Hypokinesis of myocardium or left ventricular aneurysm can lead to stasis of blood, an important component of Virchow's triad, and ultimately intracardiac thrombogenesis in the left ventricle [8]. Chronic systemic conditions predisposing to hypercoagulable states also contribute to intracavitary thrombi formation [9].

We present a unique case with a large calcified intracavitary cardiac thrombus in a young woman with obesity, a past medical history of immune thrombocytopenic purpura (ITP), and new diagnosis of systemic lupus erythematosus (SLE). Imaging presentation in our case masqueraded as a tumor, delaying the diagnosis.

## Case report

**Fig. 1:**
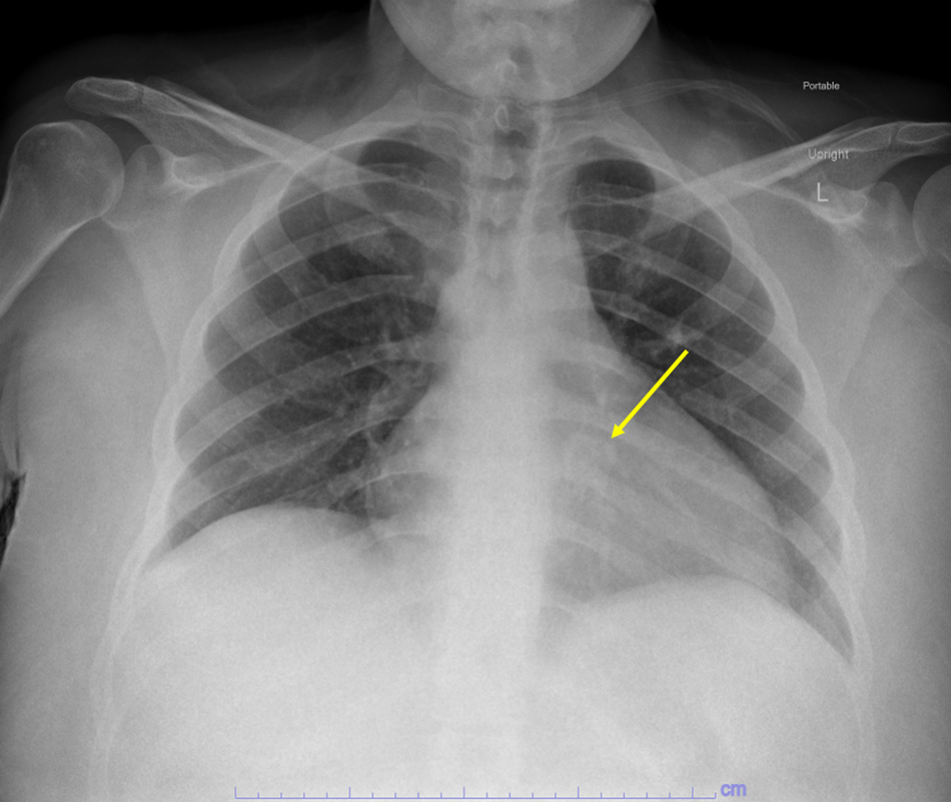
Chest Radiograph was initially reported as no acute process. However, on subsequent review, a calcified retrocardiac mass was seen (Yellow arrow) (Color version of figure is available online)

**Fig. 2:**
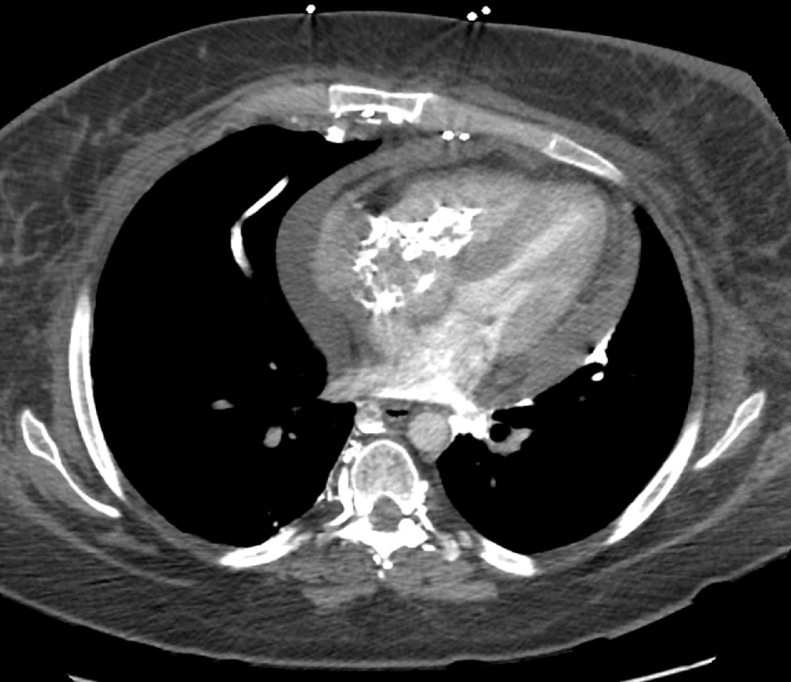
CT chest PE protocol demonstrated a large intracavitary heterogeneous irregular calcified mass extending into the right atrium, across the tricuspid valve, and into the right ventricle and pulmonary outflow tract. Additionally, small to moderate pericardial effusion was noted, with reflux of contrast in the collateral veins, suggestive of restrictive cardiac dysfunction.

This patient was a 26-year-old female with a past medical history of catastrophic antiphospholipid syndrome secondary to newly diagnosed SLE, acute on chronic ITP, hypertension, and hyperlipidemia who presented from an outside facility with shortness of breath and chest pain. She reported to her primary care physician that morning with worsening cough over the last year that became productive within the last 3 months and became painful within the last day. She described parasternal chest pain that radiated to her throat that was exacerbated by cold and smoking and a non-radiating parasternal chest pain worsened with cough. She also stated she had increasing shortness of breath upon ambulation and while lying flat. Her primary care physician sent her to her local hospital for further evaluation. Laboratory studies from the outside facility were notable for a D-dimer of 3.4 mcg/mL, hemoglobin of 18.9 g/dL, and platelet count of 130,000/uL. Chest radiograph (Fig. 1) was initially reported as no acute cardiopulmonary process. Further evaluation by computed tomography (CT) chest pulmonary embolism protocol was performed (Fig. 2), which demonstrated a large intracavitary heterogeneous, irregular, calcified mass extending into the right atrium, across the tricuspid valve, and into the right ventricle and pulmonary outflow tract. Additionally, a small to moderate pericardial effusion was noted, with reflux of contrast in the collateral veins, suggestive of restrictive cardiac dysfunction. With concern for cardiac tamponade, the patient was transferred to our institution for further evaluation by cardiology.

**Fig. 3:**
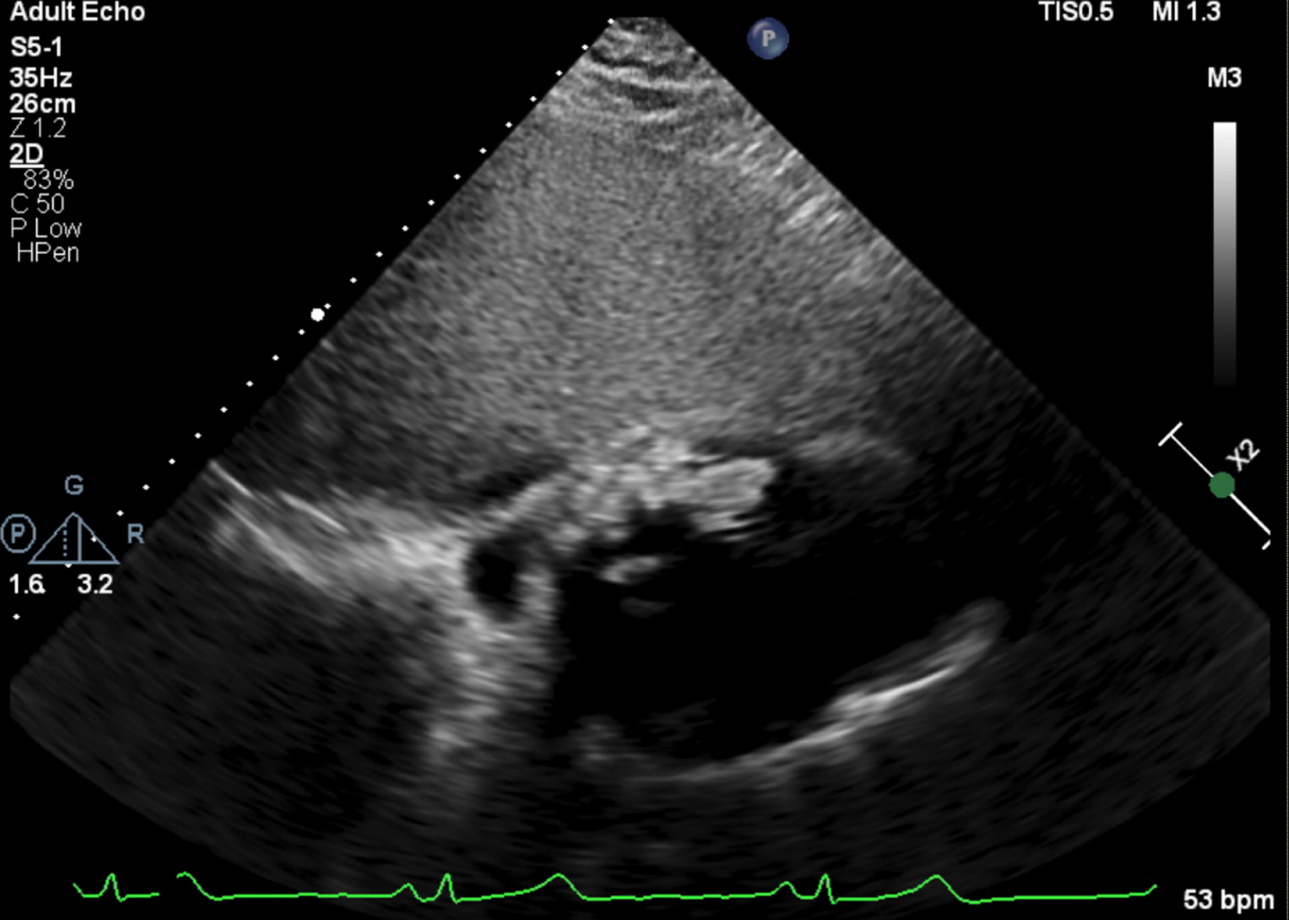
Transthoracic echocardiogram during evaluation in the emergency department confirmed the presence of a small pericardial effusion and showed significant clot burden and possible intracavitary right ventricular mass which was incompletely characterized.

After transfer from her local hospital to our tertiary University hospital emergency department, a transthoracic echocardiogram confirmed the presence of a large pericardial effusion and noted significant clot burden and possible intracavitary right ventricular mass which was incompletely characterized (Fig. 3). Normal heart rate and blood pressure in the emergency department suggested that the patient was not in cardiac tamponade, but cardiology recommended a pericardiocentesis and cardiac CT and cardiac magnetic resonance imaging (MRI).

**Fig. 4:**
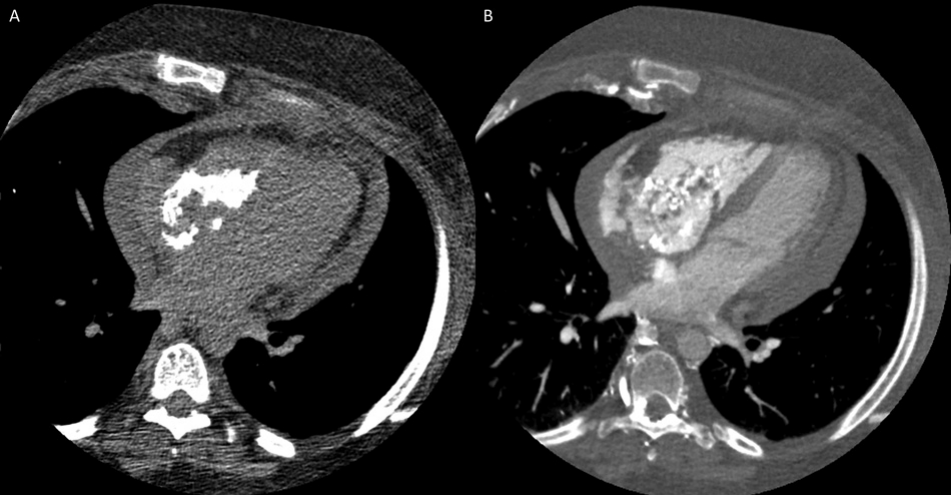
CT heart morphology revealed a heterogeneous irregular calcified mass extending into the right atrium, across the tricuspid valve. This mass had characteristics suggestive of an organized chronic thrombus, as webs of thrombus were extending throughout the right atrium and almost completely occluding the distal superior vena cava. A small pericardial effusion is also present.

**Fig. 5:**
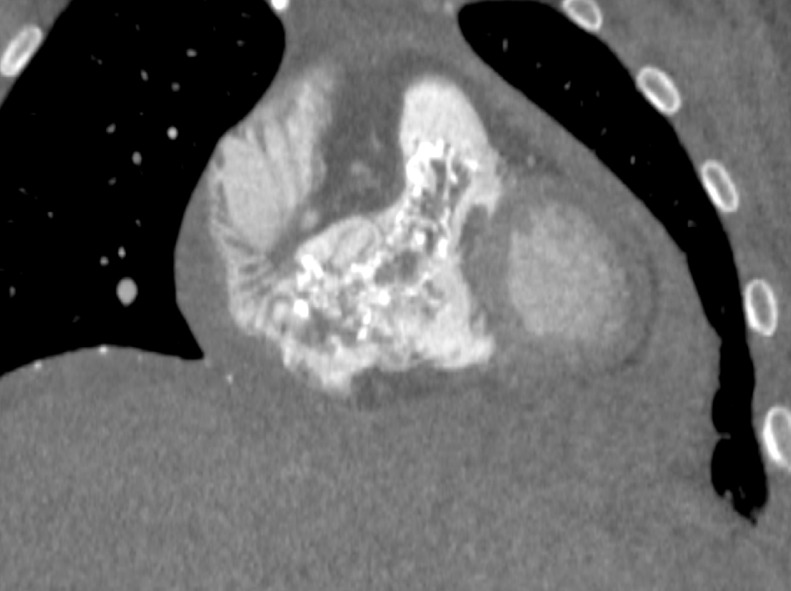
CT heart morphology, coronal reconstruction revealed a heterogeneous irregular calcified mass extending into the right ventricle and pulmonary outflow tract.

CT heart morphology (Figs. 4 and 5) revealed a heterogeneous irregular calcified mass extending into the right atrium, across the tricuspid valve, and into the right ventricle and pulmonary outflow tract. This mass had characteristics suggestive of an organized chronic thrombus, as webs of thrombus were extending throughout the right atrium and almost completely occluding the distal superior vena cava. An extensive network of collateral vessels was also demonstrated around the nearly occluded superior vena cava extending through the mediastinum, chest wall, and abdomen. A small pericardial effusion was shown, which was consistent with previous TTE findings in the emergency department.

**Fig. 6:**
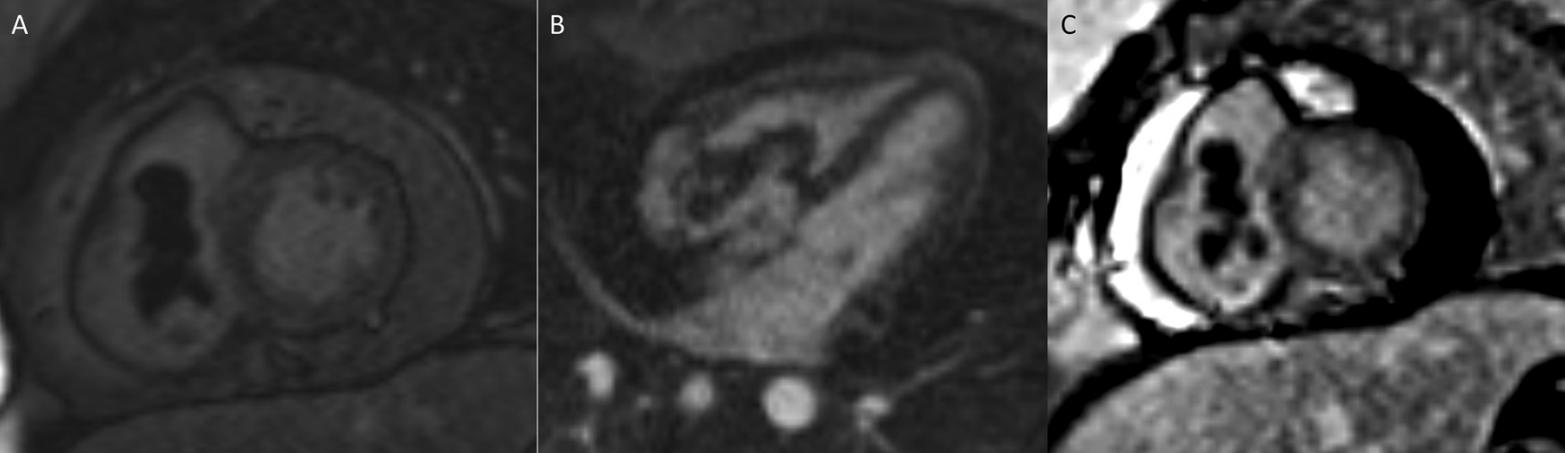
Cardiac MRI (A): Long T1 short axis demonstrated diffuse hypointense signal characteristics of this mass; (B): On first pass post contrast imaging, it was difficult to differentiate tumor enhancement from intervening blood pool. (C): Late Gadolinium Enhancement PSIR revealed no contrast enhancement. Moderate sized pericardial effusion was again noted.

Cardiac MRI (Fig. 6) further confirmed the presence of a large intracavitary right atrial mass extending into the right ventricle. This mass demonstrated a diffusely hypointense signal on long T1 sequence (Fig. 1A), and no post contrast enhancement suggesting bland thrombus, rather than a tumor thrombus. On the first pass post contrast imaging (Fig. 6B) it was difficult to differentiate tumor enhancement from intervening blood pool, but there was no contrast enhancement on delayed contrast enhanced sequences (Fig. 6C).

Further evaluation with CT of the abdomen and pelvis was unremarkable other than presence of ascites, which we attribute to cardiac dysfunction.

**Fig. 7:**
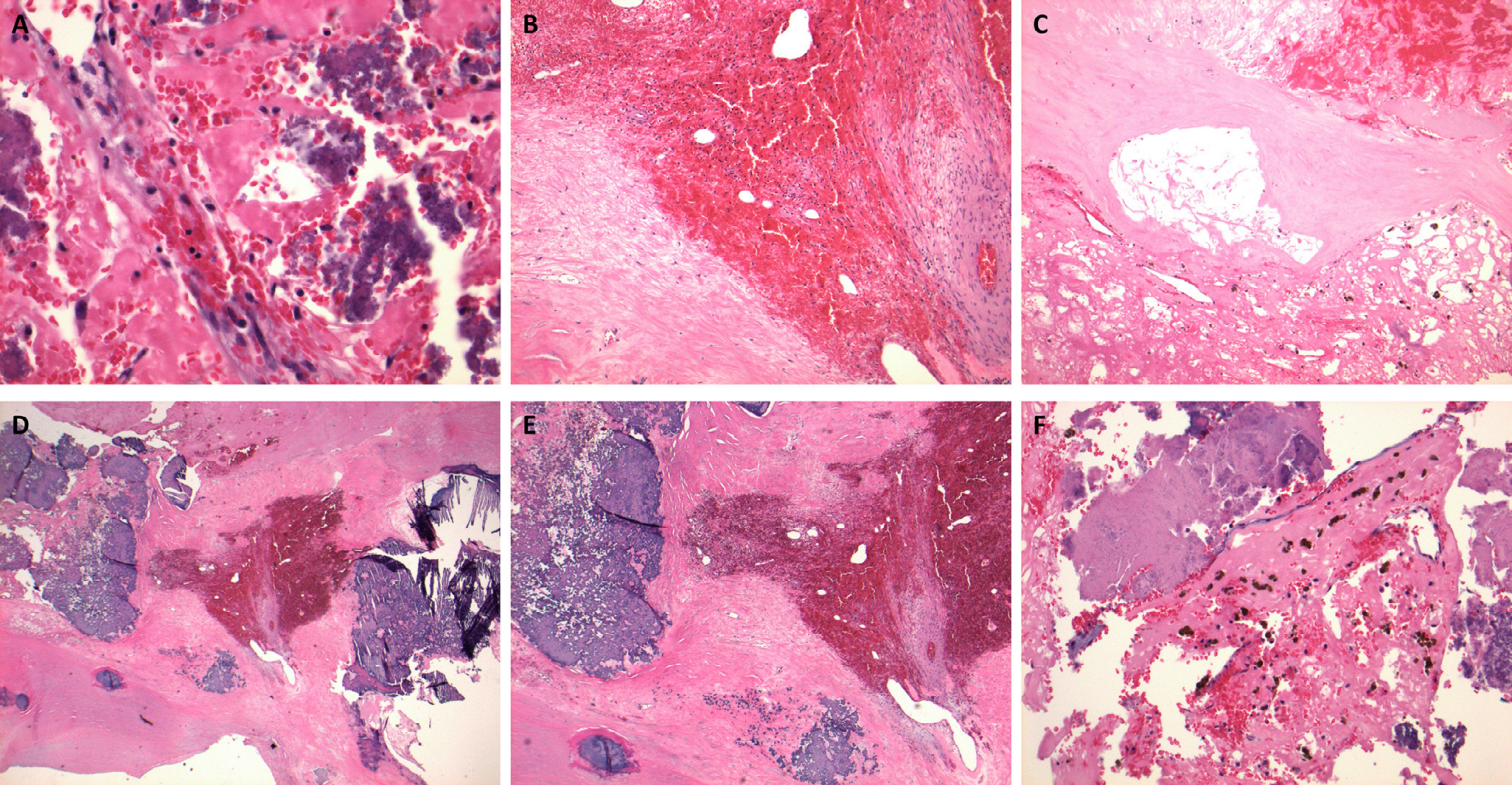
Hematoxylin and eosin (H&E) sections (B) & (E)) show the lines of Zahn which are layers of red blood cells alternating with platelets and fibrin. The lines of Zahn are characteristic of thrombus formation in the context of pulsatile blood flow. (A): Organizing thrombus with calcification (400x) (B): Organizing thrombus with fibrovascular tissue (100x). (C): Organizing thrombus with fibrous tissue and scattered hemosiderin-laden macrophages (100x). ) (D): Organizing thrombus and fibrous tissue with extensive calcifications (20x) (E): Organizing thrombus and fibrous tissue with extensive calcifications (40x). (F): Organizing thrombus, fibrous tissue, calcifications and scattered hemosiderin-laden macrophages (100x).

The patient had a complicated hospital course, as she continued to be managed at our facility for her hematological and rheumatological diseases. A successful pericardiocentesis removed 440 cc of serosanguinous fluid. Cardiothoracic surgery was consulted for surgical management of the patient's intracavitary mass. They emergently resected the 8.7 × 7.5 × 3.0 cm multi-lobulated mass that was adherent to the endocardium throughout. The differential diagnosis of the mass included thrombus versus cardiac tumor of unknown origin. Due to the extensive nature of this mass, a tricuspid valve replacement was indicated for this patient and was successfully performed using a 31 mm St. Jude Epic porcine bioprosthesis. The pathology report confirmed that the specimen was consistent with an organized thrombus with valve leaflets diffusely covered by friable, loosely adherent calcifications (Fig. 7).

Unfortunately, the patient clinically declined after cardiac surgery. She continued to have respiratory failure eventually requiring tracheostomy and had wound complications. She then had failure to thrive secondary to candida fungemia and vancomycin resistant enterococcus bacteremia, acute kidney injury requiring continuous renal replacement therapy, anemia, continued thrombocytopenia, marginally controlled diabetes mellitus, and cytomegalovirus viremia. After a complicated 72-day hospital course, she succumbed to her illnesses surrounded by her family.

## Discussion

Intracavitary cardiac thrombi are rare manifestations that can be precipitated by underlying cardiac disorders [4]. Here, we present a unique case of massive, calcified intracavitary thrombus extending from the superior vena cava to the right ventricular outflow tract, likely due to chronic hypercoagulable states secondary to ITP and SLE. The mainstay of treatment for intracavitary thrombi includes anticoagulation, which was started for this patient immediately upon admission. Definitive treatment for this patient included thrombectomy and tricuspid valve replacement per cardiothoracic surgery. Pathology reports confirmed the contents of this patient's mass to be an organizing thrombus with extensive calcifications.

Differential diagnoses of intracavitary cardiac masses include primary cardiac tumors, cardiac metastases, anatomic variants, infectious and non-infectious vegetations, and intracardiac thrombi [6,[10], [11], [12], [13], [14], [15]].

Benign tumors are frequently cardiac myxomas and the rare primary malignant tumors are frequently sarcomas [16,17]. Intracardiac thrombi may develop secondary to acute myocardial infarction, or other underlying cardiac pathologies [4]. Diagnostic clues are derived from the combination of cardiac imaging modalities, especially in patients with multiple comorbid factors.

This unique case of massive, calcified intracavitary thrombus initially presented with shortness of breath and chest pain. Two specific American College of Radiology (ACR) Appropriateness Criteria variants for this patient that might be applied include ones found under the clinical topics entitled “Chronic Chest Pain-Noncardiac Etiology Unlikely: Low to Intermediate Probability of Coronary Artery Disease (this is the link for variant 1 https://acsearch.acr.org/docs/69337/Narrative/) and “Dyspnea–Suspected Cardiac Origin” and the variant 5 entitled “Dyspnea due to suspected pericardial disease. Ischemia excluded.” (this is the link for variant 5 https://acsearch.acr.org/docs/69407/Narrative/).

According to the most recent ACR guidelines, imaging modalities deemed “usually appropriate” are a chest radiograph and CTA chest with IV contrast in the setting of acute, nonspecific chest pain. For a suspected pericardial origin of dyspnea, it is usually appropriate to use TTE, chest radiograph, MRI heart function and morphology (with or without IV contrast), CT heart morphology and CTA chest with IV contrast to further investigate the patient's symptoms [18].

Cardiac CT produces high quality images with excellent spatial resolution faster than a cardiac MRI. The ability to use electrocardiographic gating helps define lesion margins while minimizing motion-related artifact [19]. While cardiac CT was deemed inappropriate for initial evaluation of a cardiac mass, it was found to be appropriate for evaluating a cardiac mass when other imaging modalities have proved to be inadequate and for evaluating pericardial anatomy [20]. On CT imaging, a bland intracavitary thrombus appears as a hypodense intraluminal structure. Differentiating an intracardiac tumor from a thrombus requires contrast enhancement. A mass that lacks of enhancement strongly suggests a bland thrombus, thus decreasing the suspicion for primary cardiac tumors [17,21].

Cardiac MRI was deemed appropriate for the evaluation of both cardiac and pericardial masses [22]. An advantage to cardiac MRI over CT is no exposure to radiation, but this modality is much more expensive and time-consuming than cardiac CT. On T1 and T2 weighted MRI sequence, an acute thrombus has a high signal intensity while a chronic thrombus has a low T2 signal and demonstrates flow voids [23]. A bland thrombus on first pass perfusion will demonstrate a homogenous, low intensity region in every phase, as the thrombus is avascular. MRI with early gadolinium enhancement will display the thrombus as a homogenous hypointense lesion. Late gadolinium enhancement will also demonstrate low intensity and an infarct would demonstrate subendocardial enhancement.

Aggeli et. al. created a stepwise diagnostic algorithm for diagnostic evaluation of cardiac masses. Once clinical presentation and electrocardiogram are evaluated, transthoracic echocardiography was preferred as the next step in workup. If a thrombus is confirmed using ultrasound enhancing agents, they suggest that no further workup is needed, unless the thrombus involves a prosthetic valve, in which transesophageal echocardiography and cardiac CT are then indicated. Transesophageal echocardiography can also be used for the morphological characterization of atrial masses and surrounding structures, while cardiac MRI can be used for better tissue characterization of the mass itself. Since our case demonstrated an intracavitary mass extending from the superior vena cava to right ventricular outflow tract, a combination of cardiac imaging modalities (TTE, TEE, cardiac CT, and cardiac MRI) was needed to sufficiently characterize this mass as a calcified bland thrombus. Obtaining pathology results after surgical management of this mass (Fig. 7) further confirmed the diagnosis of a large, calcified intracavitary thrombus.

Intracavitary filling defect on CT is a rare finding. Differential diagnoses include benign and malignant cardiac tumors, vegetations, anatomic variants, and intracardiac bland thrombi. Cardiac MRI plays a pivotal role in differentiating intracavitary masses from thrombi, as demonstrated in our case.

## Patient consent

It was not possible to obtain verbal informed consent due to death of the patient. No identifiable patient information is shared.
